# Pan-cancer mapping of differential protein-protein interactions

**DOI:** 10.1038/s41598-020-60127-x

**Published:** 2020-02-24

**Authors:** Gizem Gulfidan, Beste Turanli, Hande Beklen, Raghu Sinha, Kazim Yalcin Arga

**Affiliations:** 1 Department of Bioengineering, Marmara University, 34722 Istanbul, Turkey; 2 Department of Bioengineering, Istanbul Medeniyet University, 34720 Istanbul, Turkey; 3 Department of Biochemistry and Molecular Biology, Penn State College of Medicine, Hershey, 17033 Pennsylvania United States

**Keywords:** Tumour biomarkers, Diagnostic markers, Prognostic markers

## Abstract

Deciphering the variations in the protein interactome is required to reach a systems-level understanding of tumorigenesis. To accomplish this task, we have considered the clinical and transcriptome data on >6000 samples from The Cancer Genome Atlas for 12 different cancers. Utilizing the gene expression levels as a proxy, we have identified the differential protein-protein interactions in each cancer type and presented a differential view of human protein interactome among the cancers. We clearly demonstrate that a certain fraction of proteins differentially interacts in the cancers, but there was no general protein interactome profile that applied to all cancers. The analysis also provided the characterization of differentially interacting proteins (DIPs) representing significant changes in their interaction patterns during tumorigenesis. In addition, DIP-centered protein modules with high diagnostic and prognostic performances were generated, which might potentially be valuable in not only understanding tumorigenesis, but also developing effective diagnosis, prognosis, and treatment strategies.

## Introduction

Deciphering the changes in the protein interactome is mandatory to reach a systems-level understanding of tumorigenesis. Additionally, physical interactions among proteins influence cellular pathways in all living organisms, and mediate diverse physiological processes^1^. On the other hand, protein-protein interactions (PPIs) are often context-specific, depending on the cellular environment, tissue type, and other dynamically changing phenotypical conditions^2,3^. Using various high-throughput proteomics approaches, over 240,000 PPIs between more than 20,000 human proteins have been discovered to date^4^ and proteome-scale map of human interactome networks are available^5–7^.

Cancer is a disease caused by abnormal cell growth due to genetic alterations in specific genes with increasing prevalence and mortality rate in most types. Therefore, it is crucial to understand the molecular mechanisms underlying cancer for early diagnosis, as well as estimation of prognosis and determining the most suitable drug treatment for cancer patients. Genomics studies have progressed towards comprehensive molecular characterization of human cancers, revealing an expanded cancer gene landscape, and defining a subset of the proteome that is intimately associated with a cancer type^8,9^. By means of comprehensive and coordinated efforts, such as The Cancer Genome Atlas (TCGA)^10^, Human Protein Atlas (HPA)^2^, and Genotype-Tissue Expression (GTEx) consortium^11^, the genome-wide expression of individual genes can be explored in different tissues and cancers. The correlations between mRNA and protein levels substantially increases the value of these extensive expression resources, allowing the use of genome-wide transcriptomic data as a proxy for prediction of protein levels^12^.

The current studies which have begun with the wide-ranging examination of alterations in cancer genes continue to understand how interactions among these genes can cause tumorigenesis^13–15^. To get a better grasp of the molecular mechanisms that discriminate a specific cancer type from other phenotypes, we ought to consider the changes in the interaction patterns of proteins, and thus obtain a differential view of human protein interactome among the phenotypes. This “differential interactome” approach leads to the identification of protein-protein interactions (PPIs) that are activated or repressed in each phenotype relative to the others. The power of the differential interactome approach has been effectively illustrated in ovarian cancer^13^ and breast cancer^16^. This approach enabled the estimation of the probability distributions for any possible co-expression profile of gene pairs (encoding proteins interacting with each other) among phenotypes and the determination of the uncertainty of whether or not a PPI is encountered in the phenotype of interest^13,16^.

TCGA represents a comprehensive resource to accelerate our understanding of cancer. The availability of the clinical survival metadata included in the TGCA database allows the association of clinical outcomes with genome-wide expression patterns of protein-coding genes. In our present study, we investigated the TCGA transcriptome and clinical data from 6876 individuals and used a systems-level approach to integrate these data with the human protein interactome network^4,6^ in order to analyze and compare the differential interactome of 12 different types of cancer (breast invasive carcinoma, colon adenocarcinoma, head and neck squamous cell carcinoma, renal clear cell carcinoma, renal papillary cell carcinoma, hepatocellular carcinoma, lung adenocarcinoma, lung squamous cell carcinoma, prostate adenocarcinoma, stomach adenocarcinoma, thyroid carcinoma, and uterine corpus endometrial carcinoma) with adequate sampling in both healthy and tumor groups. These analyses provided the characterization of differentially interacting proteins (DIPs) representing significant changes in their interaction patterns during a transition from a normal to tumor phenotype and therefore differentially relevant in the phenotype of interest. The analysis showed that a certain fraction of proteins was differentially interacting in cancer cases and had an impact on the overall patient survival. We also identified candidate protein modules with high diagnostic and/or prognostic performance, which might be useful in understanding tumorigenesis, development of novel diagnostic tools, and improvement of treatment strategies in several cancer types.

## Results

### Defining the differential interactome

**Figure 1:**
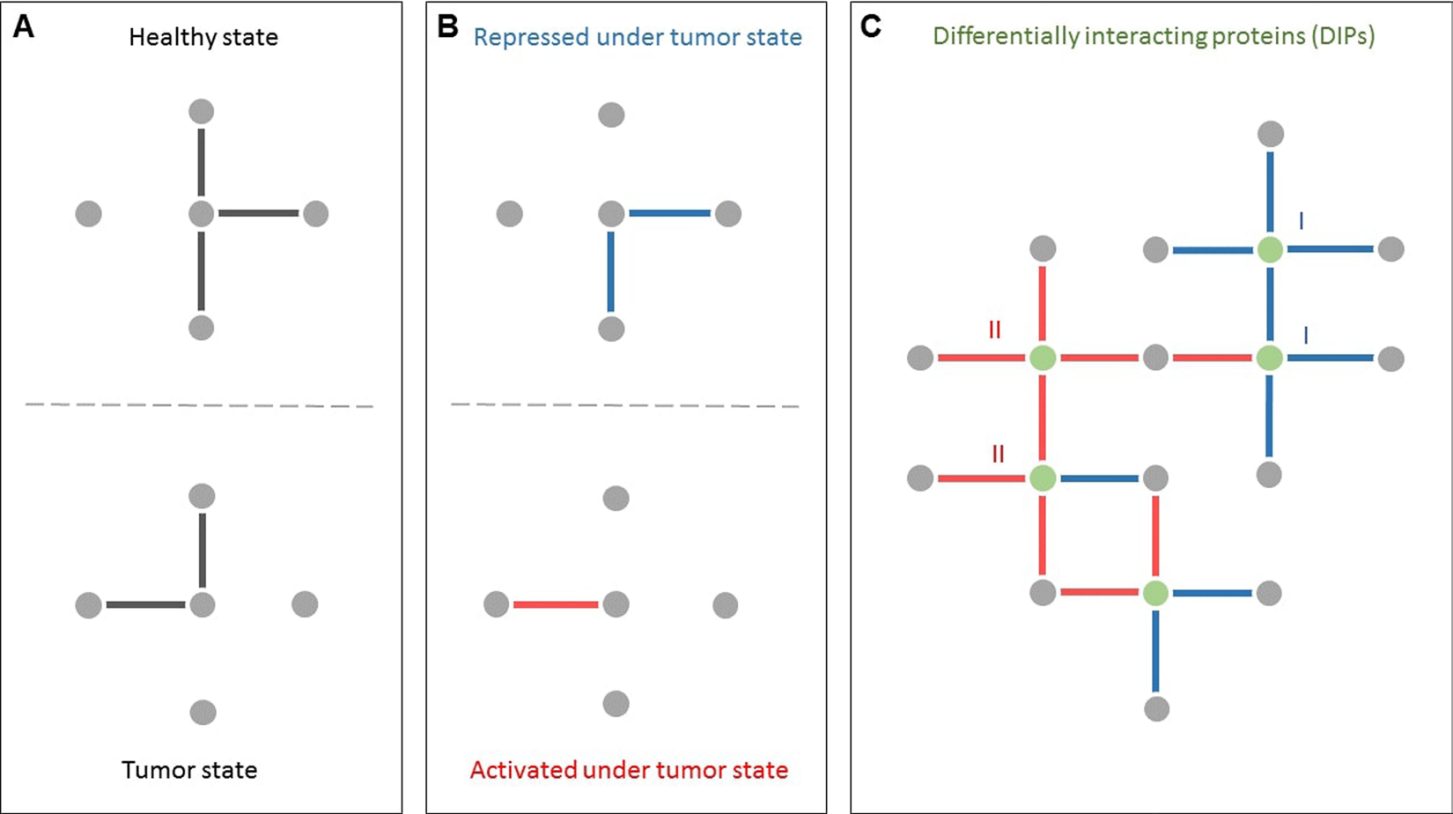
Schematic overview of differential interactome analysis between two phenotypes. (**A**) Highly probable protein-protein interactions (PPIs) in each phenotype. (**B**) Comparative analysis of interactome profiles of the phenotypes leads to the identification of differential PPIs, which are categorized into two groups as repressed or activated under the phenotype of interest (e.g., diseased phenotype). (**C**) Differentially interacting proteins (DIPs) representing significant changes in their interaction patterns during the transition between the phenotypes.

Understanding the molecular mechanisms that discriminate among phenotypes requires the estimation of the active PPIs in each phenotype (Fig. 1A). The comparative analysis of the changes in the interaction patterns of proteins will result in a differential view of human protein interactome, which consists of differential PPIs (dPPIs) with significantly different prevalence among the phenotypes. These include “activated PPIs” showing a significantly higher prevalence in the phenotype of interest (for instance, a specific cancer) compared to other phenotypes (for instance, healthy state), as well as “repressed PPIs” having a remarkably lower prevalence in the phenotype of interest (Fig. 1B).

Here, we define “differentially interacting proteins (DIPs)” as those representing significant changes in their interaction patterns during a transition from a phenotype to another phenotype and classified them into two distinct classes: Proteins exhibiting (i) an excessive repression pattern (Class I) and (ii) an excessive activation pattern (Class II) in their interactome under the phenotype of interest (Fig. 1C).

### Estimation of the differential interactome in human cancers

**Figure 2:**
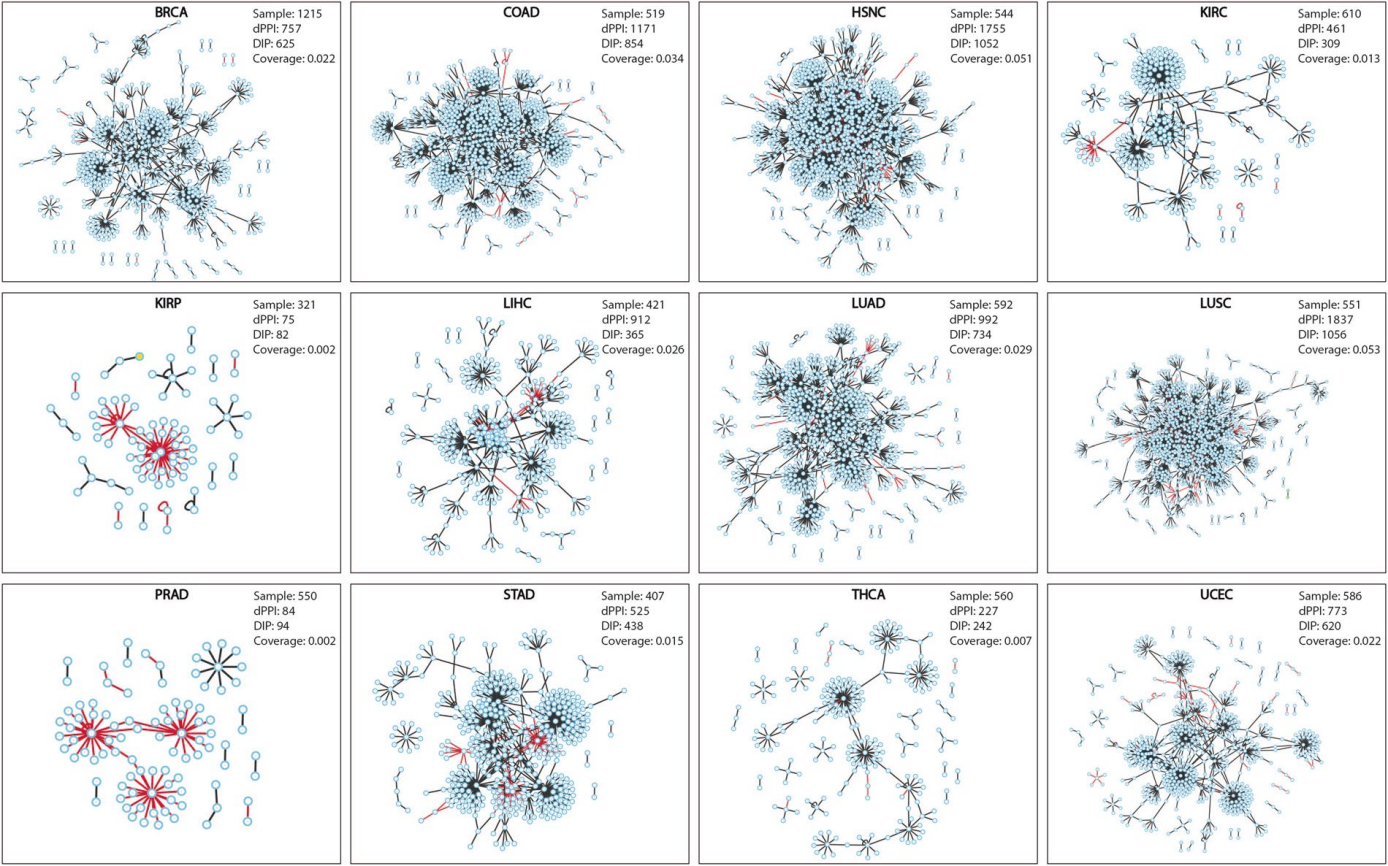
Differential interactome networks in 12 human cancers. Differential interactome network was constructed around dPPIs for each cancer. Red edges represent repressed interactions; black edges represent activated interactions.

**Figure 3:**
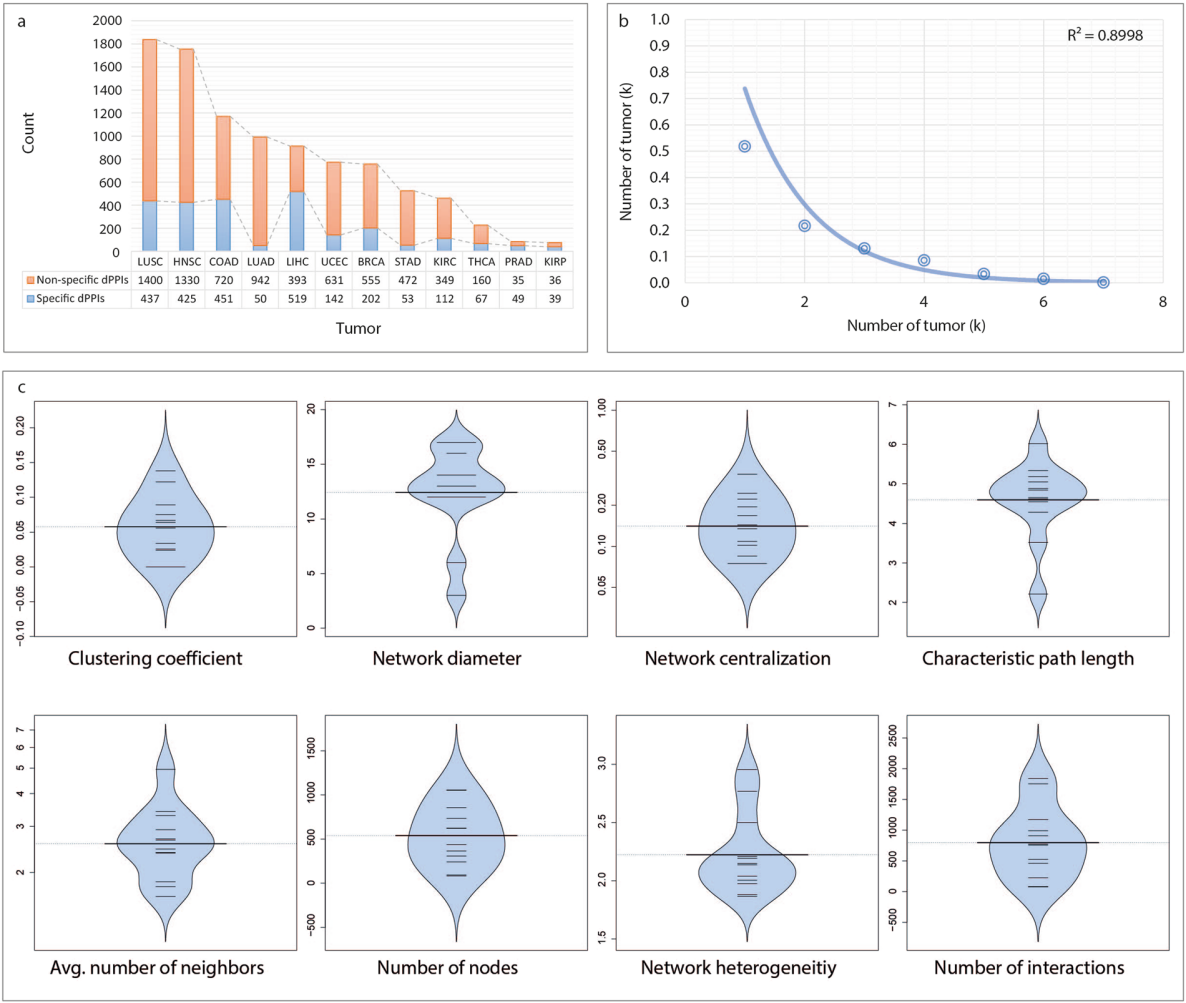
Differential interactome in human cancers. (**a**) Bar chart showing the numbers of interactions being specific and non-specific to the cancer types and their count ratio in the 12 different cancer types. (**b**) Graph indicates the prevalence of dPPIs in different cancers. (**c**) Topological characteristics of differential interactome networks. Bean plots represent the distribution of topological metrics (i.e., clustering coefficient, network diameter, network centralization, characteristic path length, average number of neighbors, network heterogeneity, number of nodes, and number of interactions) across constructed differential interactome networks. The individual observations are shown as small lines.

The RNA-sequencing derived transcriptomic data from TCGA was considered as a proxy for prediction of protein levels^12^ and the differential interactome methodology^13,16^ was recruited for estimation of highly probable PPIs in each state and identification of dPPIs. For this purpose, we investigated transcriptomic data for 12 cancers with adequate number of samples (N > 30) in both healthy and tumor groups, and a total of 6,239 tumor samples and 637 matched normal samples were analyzed (Table 1). Previously, it has been shown that the probability estimates (q values) follow a normal distribution through Lilliefors corrected Kolmogorov Smirnov test^13^. Considering a 99.5% confidence interval (i.e., p < 0.05), PPIs with q values >0.90 or <0.10 were considered as significant. Then, these PPIs were further filtered taking into consideration the protein expression levels provided by HPA^2^. For each cancer, the PPIs associated with proteins expressed at a detectable level (either high, medium, or low) in the corresponding cancer were identified as dPPIs. Differential interactome network was constructed for each cancer type around these dPPIs (Fig. 2; Supplementary Table S1). As a result, a total of 4,911 dPPIs among 2,039 proteins were detected in 12 cancers (Fig. 3a).

**Table 1:** List of human cancer types analyzed in the study.

Cancer type	Abbreviation	Number of samples	Number of normal samples	Number of tumor samples
Breast invasive carcinoma	BRCA	1215	113	1102
Colon adenocarcinoma	COAD	519	41	478
Head and Neck squamous cell carcinoma	HNSC	544	44	500
Kidney renal clear cell carcinoma	KIRC	610	72	538
Kidney renal papillary cell carcinoma	KIRP	321	32	289
Liver hepatocellular carcinoma	LIHC	421	50	371
Lung adenocarcinoma	LUAD	592	59	533
Lung squamous cell carcinoma	LUSC	551	49	502
Prostate adenocarcinoma	PRAD	550	52	498
Stomach adenocarcinoma	STAD	407	32	375
Thyroid carcinoma	THCA	560	58	502
Uterine Corpus Endometrial Carcinoma	UCEC	586	35	551

The tumor-specificity of dPPIs varied according to the cancer type (Fig. 3a). Among the cancers examined, the highest number of specific interactions belonged to LIHC with 519 specific dPPIs and a specificity rate of 56.9%. On the contrary, PRAD with 49 specific dPPIs indicated the highest specificity rate of 58.3%.

The prevalence of dPPIs decreased exponentially with increasing number of cancer types. Also, among the differential interactome 51.8% of dPPIs (2,546 interactions among 1,463 proteins) were specific to any of the cancers. On the other hand, none of the dPPIs were common for all cancer types examined, and an interaction had a common profile of at most 7 different types of cancer (Fig. 3b).

To gain structural insights into the differential interactome networks, we examined features of their network topology. Indeed, all networks exhibited features of a scale-free network with small-world property and matched the general characteristics of biological networks^17^. However, slight differences were observed in several topological parameters (Fig. 3c; Supplementary Table S2). Networks indicated similar centralization properties in terms of the clustering coefficient (0.06 ± 0.01), the average characteristic path length (4.59 ± 0.28), and the average network centralization (0.16 ± 0.02), except for KIRP that had a significantly lower average characteristic path length of 2.21 and higher network centralization of 0.34. In all the differential interactome networks, intermediate levels of network heterogeneity (2.22 ± 0.10) were observed. Both the scale free distribution and network heterogeneity levels indicated the tendency of the differential interactome networks to contain hub proteins as important components of networks.

**Figure 4:**
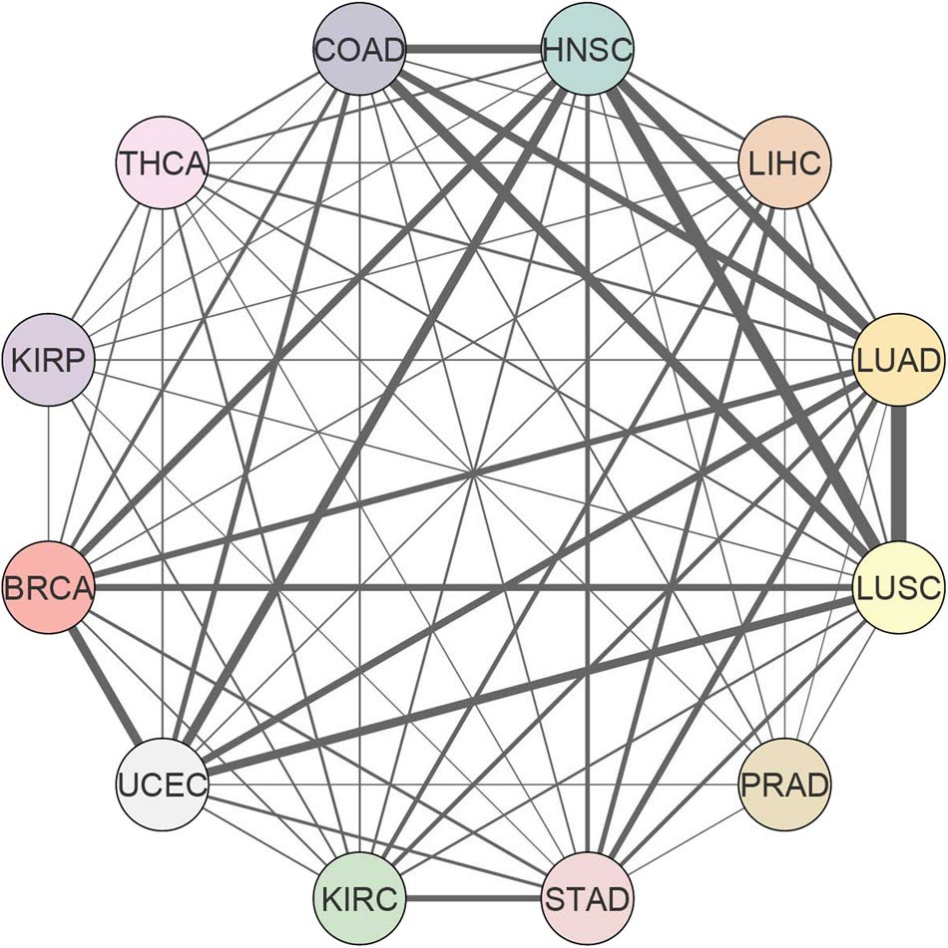
Pan-cancer analysis of the differential interactome. Similarity network showing pairwise correlations among 12 cancer types based on the Jaccard indices.

In order to further examine the similarities and differences among the interactome alterations (i.e., dPPIs) across diverse tumor types, we employed Jaccard index (JI) as the correlation metric. The quantification of pairwise similarity among cancers resulted in relatively weak correlations (JIs between 9.3 × 10^−4^ and 0.37) due to the high specificity in differential interactome networks (Fig. 4). The highest similarities were observed between LUAD and LUSC (JI = 0.37), followed by HNSC and LUSC (JI = 0.30), HNSC and LUAD (JI = 0.23), COAD and LUSC (JI = 0.23), HNSC and UCEC (JI = 0.21), BRCA and UCEC (JI = 0.21), and COAD and HNSC (JI = 0.20).

### The differentially interacting proteins in cancers

**Figure 5:**
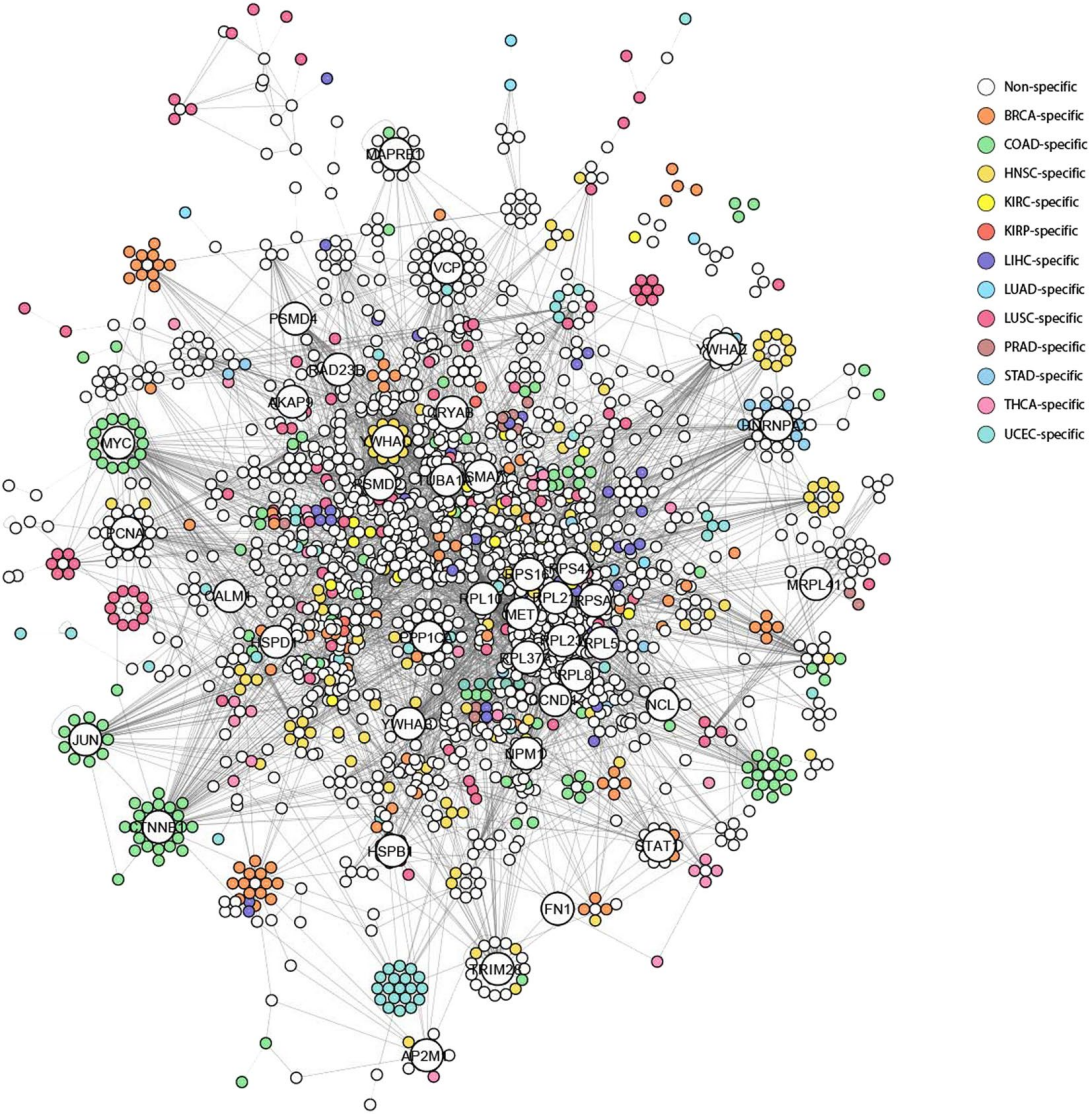
Differential interacting protein networks. Network of DIPs in 12 cancers indicates the first five DIPs having the most interactions for each cancer in larger nodes. The DIPs observed in more than one cancer type are represented in white; the cancer-specific DIPs are represented in different colors.

The analysis of the differential interactome network architecture reveals the existence of hubs, referred to as DIPs, representing significant changes in their interaction patterns during a transition from “normal” to “tumor” phenotypes, therefore differentially relevant in the tumor of interest (Fig. 1C). Topological analysis of differential interactome network of each cancer was performed to identify DIPs (Supplementary Table S3), and DIPs were classified into two distinct classes considering the repression and activation patterns of their interactome. DIPs and their interacting partners were demonstrated for each cancer also considering their cancer-specificity in Fig. 5. Among 2,039 DIPs, there is no common DIP, however, AKT1 (AKT Serine/Threonine Kinase 1), CTNNB1 (Catenin Beta 1), GRB2 (Growth Factor Receptor Bound Protein 2), HDAC1 (Histone Deacetylase 1), and HSP90AB1 (Heat Shock Protein HSP 90-Beta), which have already been associated with cancer hallmarks in COSMIC Cancer Gene Census (CGC) catalogue^18^, were the common proteins exhibiting DIP characteristics in eleven of all the cancers investigated. Furthermore, 626 DIPs were specific to any of the cancers (Supplementary Fig. S1).

The differential interactome demonstrated a network topology with modular organization, where proteins were clustered around DIPs. Therefore, we considered DIPs and their interacting protein partners as modules, referred to as DIP-centered modules, and proposed them as potential systems biomarkers for development of effective diagnosis, prognosis and treatment strategies^19^.

### Prognostic and diagnostic capabilities of DIP-centered modules

To evaluate the prognostic power of DIP-centered modules, in each cancer, the patient cohort was partitioned into low- and high-risk groups according to the expression levels of each gene presented in the module, and multivariate survival analyses and risk assessments were performed. Kaplan-Meier plots, log-rank test and hazard ratios (HRs) were employed to quantify the prognostic capabilities of modules. These analyses were conducted using TCGA RNA-seq datasets (see Methods).

**Figure 6:**
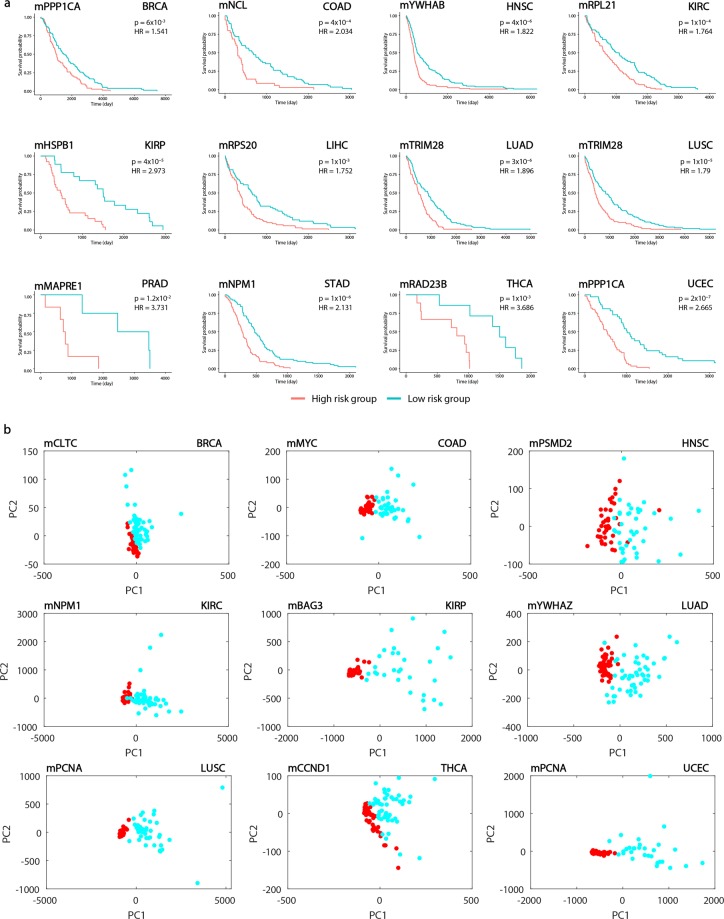
Prognostic and principal component analyses for different cancer types. (**a**) Kaplan-Meier Plots estimating patients’ survival for 12 cancer types indicating p-value and hazard ratio for each curve. (**b**) PCA plots showing the individual differences in the gene expression profiles among the cancers including at least 30 individuals in each type.

When the association of the expression levels of DIP-centered modules (i.e., DIPs and their interacting partners) with prognostic outcome was investigated through survival analyses, we observed that a total of 90 DIP-centered modules showed high impact on overall patient survival (p < 0.05 and HR > 1.3) in several tumors (Supplementary Table S4) as exemplified in Fig. 6a.

Diagnostic feature of each module was analyzed by Principle Component Analysis (PCA). Considering the most significant principle components (representing at least 80% of the total variance), sensitivity, specificity and diagnostic odds ratio (DOR) metrics were calculated. A total of 41 modules exhibited significantly high diagnostic performance (sensitivity ≥ 0.90, specificity ≥ 0.90, DOR ≥ 81) in several tumors (Supplementary Table S4) and the most significant results are depicted in Fig. 6b.

Among the DIP-centered modules exhibiting high diagnostic or prognostic performance in any of the cancers, eight modules (mNCL, mPCNA, mPSMD2, mPSMD4, mTRIM28, mVCP, mYWHAG, mYWHAZ) in LUSC, six modules (mCCT3, mNPM1, mPCNA, mTRIM28, mYWHAG, mYWHAZ) in LUAD, five modules (mPCNA, mPSMA7, mPSMB7, mPSMC3, mPSMD4) in UCEC, four modules (mCTNNB1, mPRPF19, mTRIM28, mYWHAG) in COAD, three modules (mCRYAB, mFLOT2, mMET) in KIRP, two modules (mPSMD2, mVCP) in HNSC, two modules (mCLTC, mPSMD4) in BRCA, one module (mNPM1) in KIRC indicated both diagnostic and prognostic characteristics (Supplementary Table S4).

### Association of DIPs with cancer hallmarks

Cancer cells acquire mechanisms to evade host growth suppressors and therefore tumor suppressor proteins (TSPs) as well as oncoproteins play crucial roles in cancer genome reprogramming. Hallmarks of cancer provide a comprehension of complexity of neoplastic diseases^20^. Fouad and Aanei defined seven cancer hallmarks, such as selective growth and proliferative advantage, altered stress response favoring overall survival, vascularization, invasion and metastasis, metabolic rewiring, an abetting microenvironment, and immune modulation^21^. We investigated the presence of TSPs and oncoproteins among DIPs using a list of proteins consisting of 1,217 TSPs and 803 oncoproteins^22^, and examined the participation of DIPs in molecular pathways and biological processes associated with cancer hallmarks by gene over-representation analyses. We observed that a significant number of DIP-centered modules were centralized around TSPs and oncoproteins in each cancer type (Supplementary Fig. S2). Furthermore, a large number of dPPIs highlighted pathways and processes that enable the acquisition of or maintain hallmarks of cancer including sustaining proliferative signaling, evading growth suppressors, resisting cell death, enabling replicative immortality, inducing angiogenesis, activating invasion and metastasis, deregulating cellular energetics, avoiding immune destruction, genome instability and mutation (Supplementary Fig. S3).

The known driver genes take roles through a various signaling pathways regulating main cellular processes such as determination of cell fate, cell survival, and genome maintenance^9^. By using the published driver gene information^9^, we investigated whether DIPs had the feature of driver genes and found that 13 DIPs were among 43 cancer predisposition genes, 6 DIPs were among 13 driver genes affected by amplification or homozygous deletion, and 70 DIPs were among 125 driver genes affected by subtle mutations (Supplementary Table S5).

### Druggability of the DIPs

**Figure 7:**
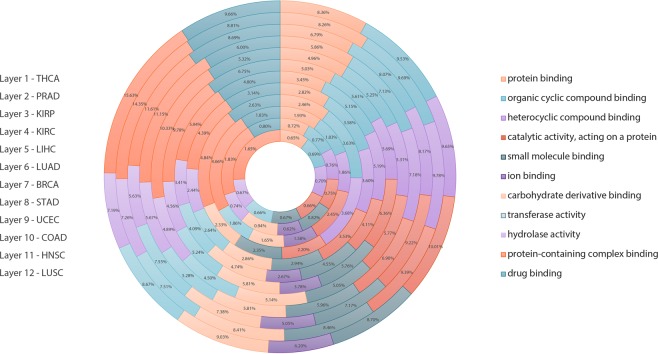
Over-representation analysis for different cancer types. The graph shows the percentages of DIPs in various molecular functions to all genes participating in these molecular functions for different cancer types. Each molecular function is represented in different color and name of the cancers are indicated on the left going from the inner to the outer layer.

**Figure 8:**
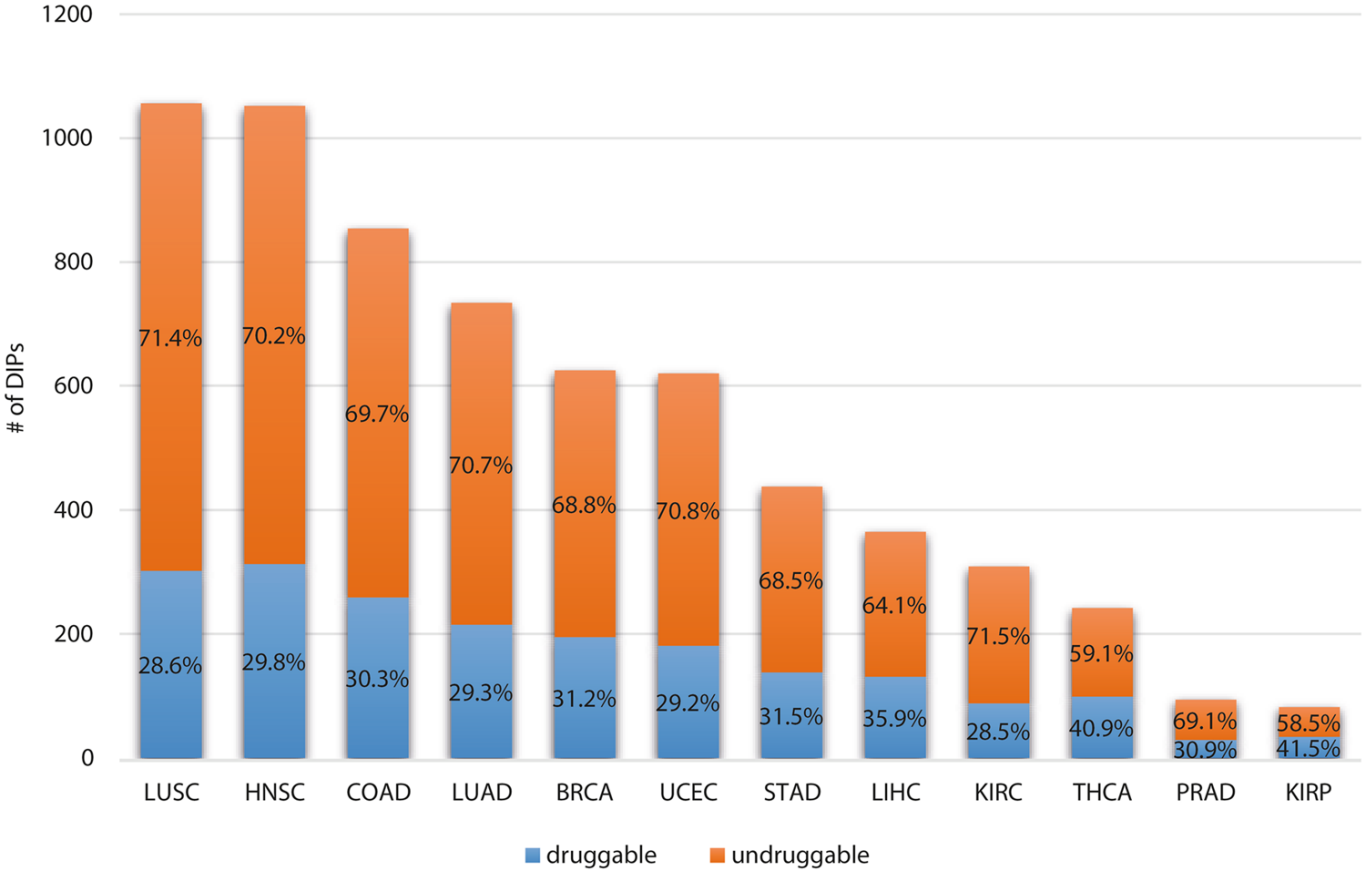
Classification of DIPs. The stacked bar graph indicates the number of DIPs, which are druggable (blue bars) and undruggable (orange bars) and their percentage for each cancer.

The druggability of the DIPs is an important issue since the cancer enabling PPIs has become promising therapeutic targets in recent years. Also taking into consideration the fact that most of the DIPs exhibit binding activities (such as protein binding, organic cyclic and heterocyclic compound binding, ion binding and drug binding) (Fig. 7), we analyzed the druggability of the DIPs. We observed that on average 32% of the DIPs were druggable. KIRP had the highest percentage (41%) of druggable proteins, while the highest number of druggable DIPs belonged to HNSC (313 proteins) amongst all cancers analyzed (Fig. 8).

## Discussion

A combination of genetic and epigenetic alterations determines the oncogenic potential of a cell through the operation of cellular networks. PPIs have crucial roles in network connectivity maintaining cancer cells growth, transmitting oncogenic signals and gaining hallmark features of cancer^23,24^. For example, downstream phosphorelay signal transduction systems were rewired by altered PPIs (such as enhanced 14-3-3 interactions with Bad, FOXO3a, and PRAS40) as a result of Akt-activating mutations^23^.

Network-based approaches can assist in understanding the differential protein interactome associated with tumorigenesis and thus can aid in unveiling the cancer mechanisms and finding therapeutic targets. Therefore, studying protein interactome is very important for the investigation of genetic alterations in the network by considering the system as a whole^25^.

This study reports the generation of dPPI networks in human cancers through the implementation of high throughput transcriptome and protein interactome data. The focused RNA-seq datasets coupled with a differential interactome methodology allowed the identification of dPPIs between normal and tumor states in 12 different human cancers. The study reveals the dPPIs in each cancer type and also provides a pan-cancer analysis of differential interactome. Although several papers on pan-cancer analysis of genomic alterations have been published^26,27^, along with development of integrated pictures of commonalities, differences and emergent themes across tumor types by research consortium such as TCGA, this is the first study to our knowledge that defined the concept of the “differentially interacting protein (DIP)” and identified DIPs through a pan-cancer analysis of differential interactome. We believe that this concept shed a light on not only the dynamic structures of cancer networks but also brings a new perspective to further network-based analyses.

The high specificity of dPPIs to the cancer types is remarkable in terms of displaying the heterogeneity in molecular mechanisms of tumorigenesis. When we examined the similarities and differences among the interactome alterations across diverse tumor types it turned out that no general protein interactome profile was applicable to all cancers. Among the differential interactome, none of the dPPIs were common to all types of cancer examined, and more than half of the dPPIs were specific to any of the cancers. Moreover, similarity among cancers was represented with relatively weak correlations.

In our analysis, the heterogeneity was also represented in topology of differential interactome networks. The network heterogeneity, which is measured by the variance of the degree distribution, is considered as an important topological metric to define cooperative behavior within the networks and to reflect the tendency of the networks to contain hub nodes. Recently, the impact of different levels of network heterogeneity on the fate of cooperation in social networks was studied from evolutionary game theory perspective^28^. These results implied that the best evolutionary outcome was associated with intermediate levels of heterogeneity in the society. Therefore, the presence of an optimal range of heterogeneity level that maximizes the resilience of the society was suggested^28^. Analogous to social networks in all differential interactome networks, intermediate levels of network heterogeneity (2.22 ± 0.10) were observed, indicating the cooperative re-programming in protein interactome in cancers. By comparison with random networks having the same size of the cancer networks, it was shown that the topological features of cancer networks were not by chance, since the network parameters of the cancer networks were quite different than those of random networks and the differences were statistically significant (Supplementary Fig. S4).

Both the scale free distribution and network heterogeneity levels pointed out the tendency of the differential interactome networks to contain hub proteins (DIPs), and functional over-representation analyses of DIPs presented their vigorous associations with classical hallmarks of cancers and characteristics that are intricately associated with cancers (Supplementary Fig. S3). These observations paved the way for the hypothesis that DIPs transmit pathophysiological cues along molecular networks to promote tumorigenesis, tumor progression, invasion, and/or metastasis. Therefore, special attention ought to be given to hub proteins (DIPs) and the modular structures around these hubs (DIP-centered modules) to evaluate their capabilities in development of effective diagnosis, prognosis and treatment strategies.

It is a challenging task to develop highly accurate and robust biomarkers considering the complexity of the molecular mechanisms behind these pathologies. Traditionally, biomarker discovery is concentrated on molecular biomarkers such as a single gene, protein or metabolite; however, this way of thinking has not been successful since the development and progression of cancers are mostly caused by coordinated action of a group of biological entities, rather than from the malfunction of an individual molecule^29,30^. Therefore, we propose tumor-specific DIP-centered modules as potential systems biomarkers for precise diagnosis and prognosis of cancers considering their high prognostic and diagnostic performances in several tumors demonstrated by survival and stratification analyses (Fig. 6).

In recent years, targeting PPI interfaces as a treatment strategy has become a reality^31^. Promising results were obtained for several compounds as potential inhibitors of PPIs, for example, as an inhibitor of the CCR5/gp120 interaction, Maraviroc is currently available on the market as anti-HIV drug^23^. Such efforts demonstrate the feasibility of targeting cancer-enabling PPIs for treatment of cancers. The present differential interactome analysis showed that most of the DIPs exhibit binding activities and 32% of the DIPs were druggable. Considering that a large number of PPIs are involved in driving tumorigenesis, we expect that interception of critical DIPs (and their modules) may disable essential survival mechanisms in cancer cells. Therefore, we propose these PPI interfaces as potential targets for anticancer therapeutic discovery and development^32^. Moreover, these specific dPPIs can be appraised in drug repositioning for development of cancer therapy^16,33,34^.

## Methods

### Gene expression data

Gene expression profiles were obtained from the Cancer Genome Atlas (TCGA)^35^ for 12 different types of cancer having at least 30 normal and tumor samples gathered. RNA-sequencing (RNA-seq) reads (normalized as FPKM) were collected from a total of 6,239 tumor tissue samples and 637 matched normal tissue samples for all cancers (Table 1), and employed in identification of differential interactome.

### Protein-protein interactions data

Human protein interactome containing 35,688 physical and experimentally detected PPIs among 8,570 human proteins were collected from the BioGRID database (MV-Physical-3.4.161)^4^. Filtering the interactome dataset with proteins encoded by the genes, which were represented in the transcriptome datasets from TCGA, resulted in a network of 34,603 PPIs among 8,322 proteins.

### Identification of differential interactome

Differential interactome algorithm^13^ was implemented in R (version 3.6.1) (https://www.R-project.org/) and applied to gene expression profiles in each cancer type to estimate the relative observation frequency (q-value) for each PPI, as previously described^13^. Briefly, the genes were categorized into three levels (−1, 0, 1) according to their expression levels within each sample (i.e., −1 corresponds to low expression, 0 corresponds to average expression level, and 1 corresponds to high expression level). According to that three-level formulation, 9 possible gene expression states were defined (i.e., [0 0], [0 1], [0 −1], [1 0], [1 1], [1 −1], [−1 0], [−1 1], [−1 −1]) for each interacting protein pair. For each state, the number of times (N_0_) that the state appeared in control group and the number of times (N_1_) that it appeared in tumor group were counted. Considering the possible imbalance between the sample sizes of control and tumor groups, the counting parameters were normalized taking into account the total sizes of control (N_C_) and tumor (N_T_) groups, which are the maximum possible values of N_0_ and N_1_, respectively). The estimation of the probability that any state is encountered in tumor condition was represented by q value as follows:

$${\rm{q}}=\frac{\frac{{{\rm{N}}}_{1}}{{{\rm{N}}}_{{\rm{T}}}}}{\frac{{{\rm{N}}}_{0}}{{{\rm{N}}}_{{\rm{C}}}}+\frac{{{\rm{N}}}_{1}}{{{\rm{N}}}_{{\rm{T}}}}}$$

In the current formulation, the PPIs ensuring the following criteria were considered in further analyses; (1) having q-value lower than 0.10 (significantly repressed in tumor phenotype) or higher than 0.90 (significantly activated in tumor phenotype), (2) having a normalized observation frequency either in normal or tumor phenotype higher than 20%. Then, these PPIs were further filtered taking into consideration the pathology data presented by the Human Protein Atlas (HPA) (version 19)^2^, which include protein expression levels of 19,651 proteins in 20 cancer types. For each cancer, the PPIs associated with proteins expressed at a detectable level (either high, medium, or low) in the corresponding cancer were identified as “differential PPIs (dPPIs)”, and evaluated in further analyses.

The hub proteins of the differential interactome network of each cancer, which represent significant changes in their interaction patterns during a transition from “normal” to “tumor” phenotypes, were named as differentially interacting proteins (DIPs). DIPs were distinguished into two classes based on their interaction patterns: Class I represents the proteins having repressed interactions under tumor state, whereas the proteins having activated interactions under tumor state is grouped as Class II.

DIPs together with their interacting protein partners were assigned as DIP-centered modules and the statistical significance of each module was estimated by Kruskal–Wallis test comparing the observation frequencies of dPPIs among tumor and control states. Modules with p-value < 0.05 were considered significant.

### Gene set over-representation analysis

To identify functional annotations (i.e., biological processes, molecular functions, signaling and metabolic pathways) significantly associated with the gene products, over-representation analyses were performed using ConsensusPathDB^36^. For the pathway analysis, Kyoto Encyclopedia of Genes and Genomes (KEGG)^37^ was used as the pathway database. Gene Ontology (GO) terminology^38^ was employed as the source for annotating the molecular functions and biological processes. P-values representing the significance of overrepresentations were obtained via Fisher’s Exact Test. Benjamini-Hochberg correction was used as the multiple testing correction technique and enrichment results with adjusted p < 0.05 were considered statistically significant.

### Network visualization and topological analysis

PPI networks were visualized and topological properties of the networks were identified using Cytoscape 3.4.0^39^. Topological metrics were given as mean ± SEM.

### Diagnostic performance analysis

Principal component analyses (PCA) were performed based on the TCGA originated gene expression profiles of genes encoding DIPs in each DIP-centered module. Each simulation was performed using at least randomly chosen 30 normal and 30 tumor samples and the first two principle components explaining at least 80% of total variance were considered in determination of sensitivity and specificity metrics. Simulations were repeated until the robustness in the average value of the metrics was ensured.

### Prognostic performance analysis

Survival analyses were performed according to the well-established pipeline^40,41^ using RNA-Seq data originated TCGA datasets in order to identify prognostic performance of each DIP-centered module. The subjects were partitioned into low- and high-risk groups according to their prognostic index (PI), also known as the risk score, which is the linear component of the Cox model (PI = β_1_x_1_ + β_2_x_2_ +… + β_p_x_p_, where x_i_ is the expression value of each gene, β_i_ is coefficient obtained from the Cox fitting). All analyses were carried out with *survival* package in R (version 3.6.1) (https://www.R-project.org/). The survival signatures in each group were evaluated by Kaplan-Meier plots, and a log-rank p-value <0.05 was considered as the cut-off to describe statistical significance.

## Supplementary information

Supplementary Information.

Supplementary Information 2.

## Data Availability

The source codes for the differential interactome algorithm (implemented in R, version 3.6.1) are freely available at http://sysbio.bioe.eng.marmara.edu.tr/diff-int-ome.
