# Supplementary material for: Pan-cancer mapping of differential protein-protein interactions
Source: Sci Rep. 2020 Feb 24;10:3272. doi: 10.1038/s41598-020-60127-x (PMC7039988; doi:10.1038/s41598-020-60127-x)
Supplement: Supplementary file 1 — Supplementary Information. [file 41598_2020_60127_MOESM1_ESM.pdf]

Supplementary information for Scientific Reports

Supplementary data of the article: “Pan-cancer mapping of differential protein-protein interactions”

Gizem Gulfidan [1], Beste Turanli [1,2], Hande Beklen[1], Raghu Sinha[3] and Kazim Yalcin Arga [1]*

[1] Department of Bioengineering, Marmara University, 34722 Istanbul, Turkey
[2] Department of Bioengineering, Istanbul Medeniyet University, 34720 Istanbul, Turkey
[3]Department of Biochemistry and Molecular Biology, Penn State College of Medicine, Hershey, 17033 Pennsylvania, United States
* Correspondence and requests for materials should be addressed to K.Y.A (kazim.arga@marmara.edu.tr)

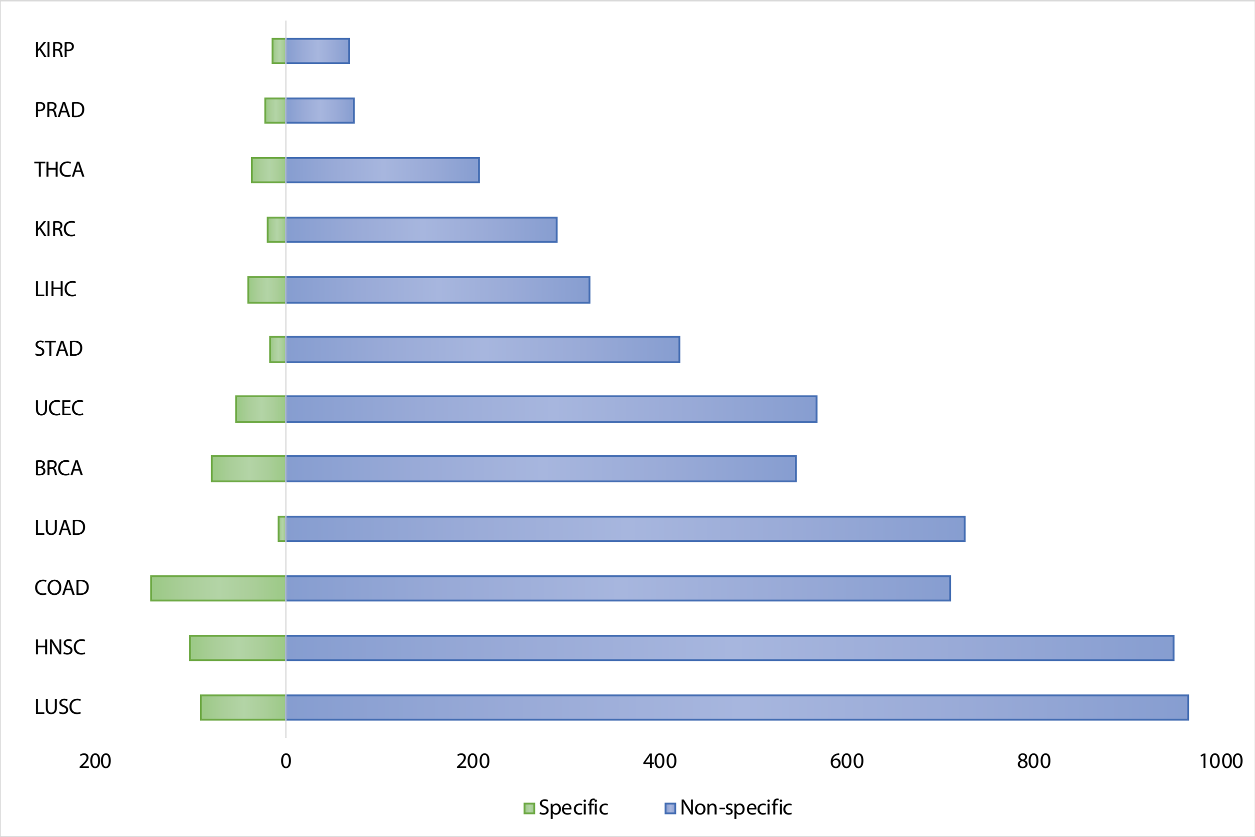

Supplementary Figure S1. The number of DIPs which were specific or non-specific to any cancer type.

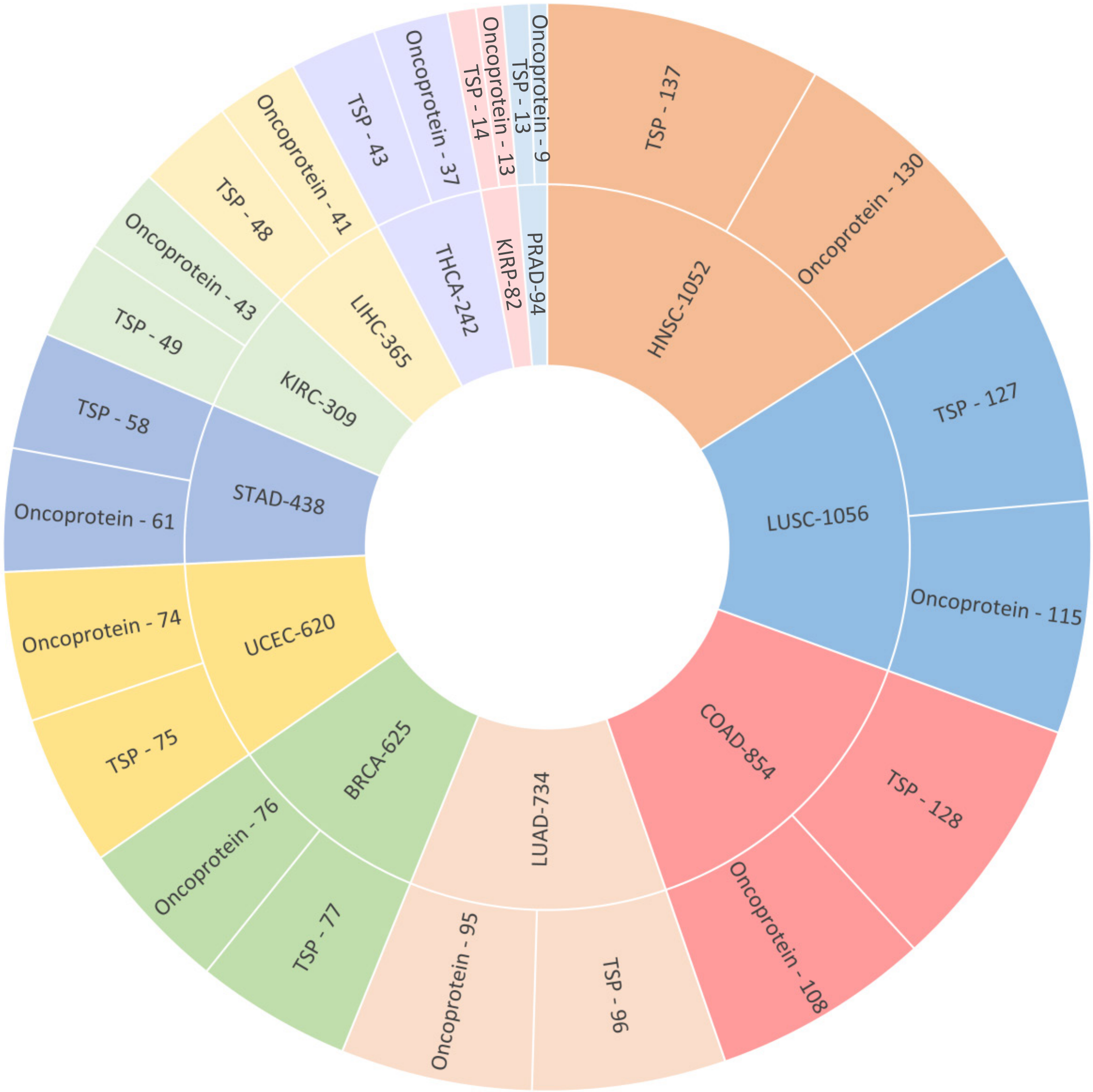

Supplementary Figure S2. The numerical distribution of all DIPs and their TSP and oncoprotein features in each cancer type.

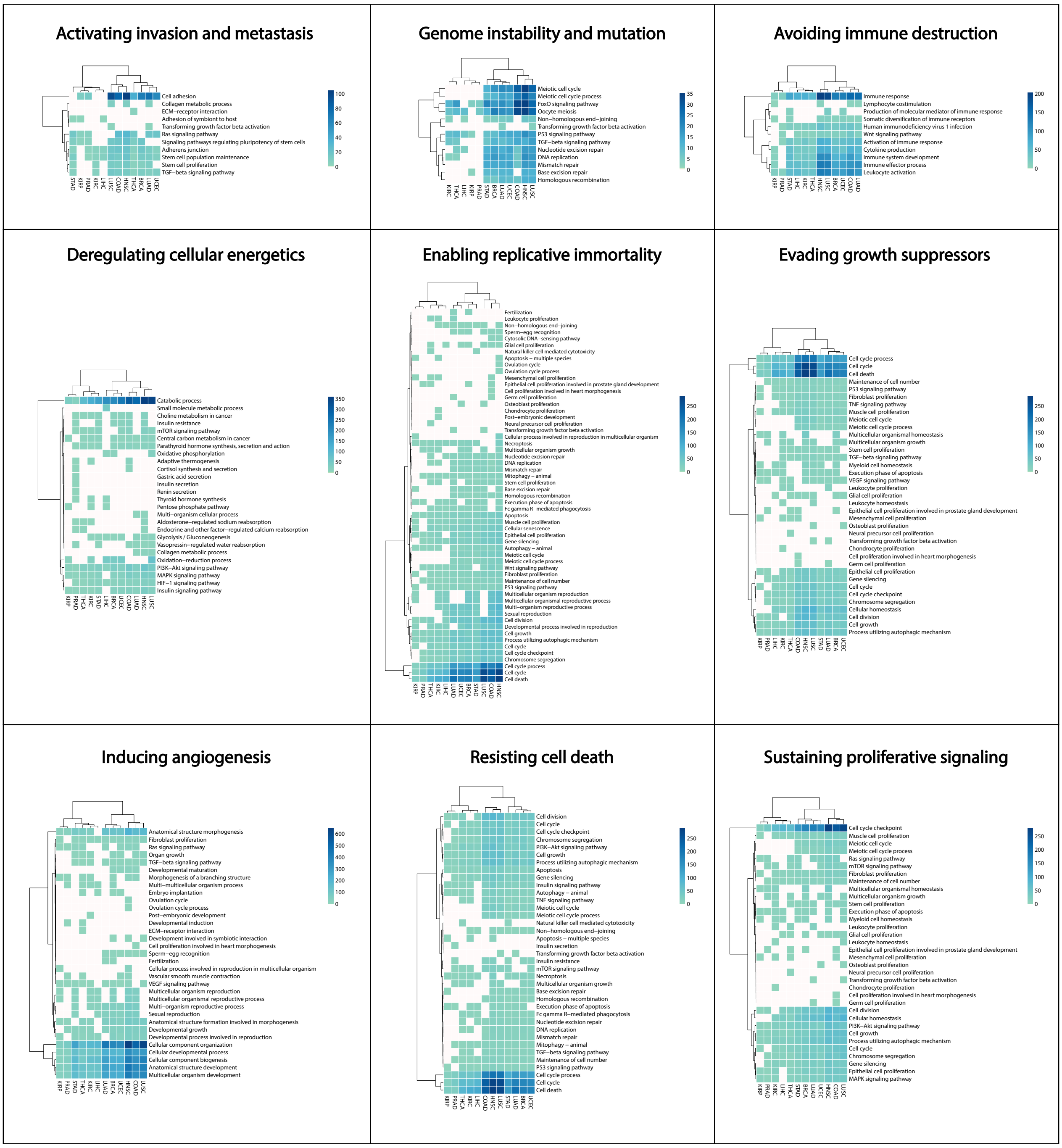

Supplementary Figure S3. Heatmap indicating cancer hallmarks. The heatmaps shows the number of genes taking part in biological process and molecular pathways for each cancer type and are classified according to known cancer hallmarks.

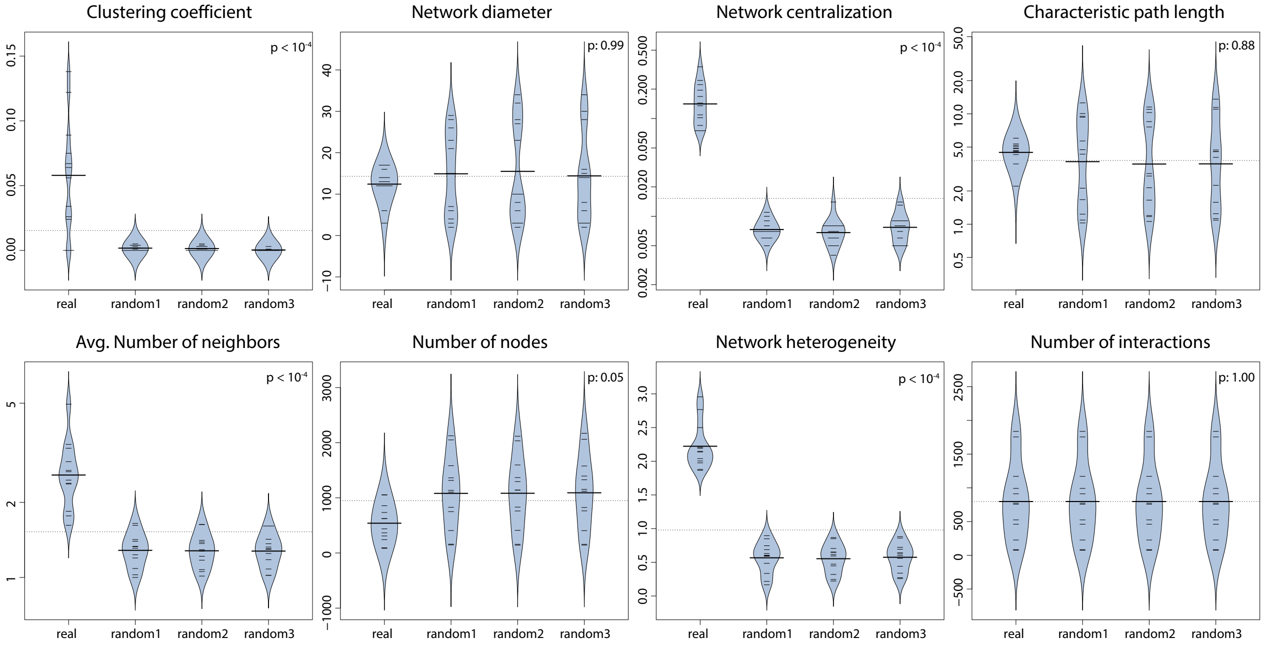

Supplementary Figure S4. The bean plots indicating a comparison between network topologies of dPPIs and various random interactions.
